# Antioxidant Activities of Extracts and Fractions from *Eupatorium lindleyanum* DC

**DOI:** 10.3390/molecules16075998

**Published:** 2011-07-19

**Authors:** Guilong Yan, Lilian Ji, Yuming Luo, Yonghong Hu

**Affiliations:** 1 College of Biotechnology and Pharmaceutical Engineering, Nanjing University of Technology, Nanjing 210009, China; 2 Jiangsu Key Laboratory for Eco-Agricultural Biotechnology around Hongze Lake, Huaiyin Normal University, Huaian 223300, China

**Keywords:** *Eupatorium lindleyanum* DC, antioxidant activity, extracts, phenolics

## Abstract

The antioxidant activities of water extract (WE), ethanol extract (EE), residue water extract (RWE) and petroleum ether (PF), ethyl acetate (EF), *n*-BuOH (BF) and water (WF) fractions of the ethanol extract from *Eupatorium Lindley* DC were investigated for the first time. Total phenolics content, DPPH radical scavenging activities, superoxide radical scavenging activities, total reduction capability, and ferrous ions chelating activities were determined for all the extracts and fractions. The results showed that all the extracts and fractions exhibited antioxidant activities with different magnitudes of potency. Among all the samples, WE and RWE exhibited the best antioxidant capacities, the BF also exhibited high antioxidant abilities in all tests except for the metal chelating activity, while the other extracts and fractions were relatively weak antioxidants. The BF had the highest total phenolics contents in all extracts and fractions, and the WE and RWE were found to be rich in tannins. Furthermore, the content of total phenolics showed good correlation with DPPH radical scavenging activity, superoxide anion radical scavenging activity, and the reducing power. Phenolic composition of all the extracts and fractions was identified and quantified by HPLC. The results indicate that the extracts of *E. Lindley* DC might be a useful potential source of natural antioxidant ingredients.

## 1. Introduction

It is well known that reactive oxygen species (ROS) can cause various diseases, such as cancer, cardiovascular disease, inflammatory injury and neurodegenerative disorders [1,2,3]. Thus, much attention has been paid in recent years to finding antioxidants against oxidative damage induced by free radicals. In addition there are health risks in the chronic consumption of synthetic antioxidants [4], thereby increasing interest in screening for natural antioxidants from plants and other bioresources [5,6,7,8,9].

*Eupatorium lindleyanum* DC is a traditional Chinese herb. Its aerial part, called ‘Yemazhui’, is mainly used for the treatment of chronic bronchitis, tracheobronchitis, hypertension, cold and fever, cough with sputum, headache, tonsillitis, bacillary dysentery, *etc* [10]. Ji *et al.* [11] further reported the antimicrobial effects of a decoction of *E. lindleyanum* DC. Phytochemistry studies [10,12,13,14,15] have shown that *E. lindleyanum* DC contained volatile oil, flavonoids and alkaloids, coumarins, sesquiterpenes and esters, and within those compounds, some are well-known antioxidants. However, there are no relevant studies on the antioxidant activities of this herb. Therefore this paper aimed to address the study of the antioxidant activities of the extracts from *E. lindleyanum* DC and the derived fractions. Furthermore, the specific phenolic compositions of the extracts and fractions were also determined.

## 2. Results and Discussion

### 2.1. Total Phenolics Contents

Many studies have revealed that the antioxidant activity of plant products is mainly due to phenolic compounds [16,17]. The content of the total phenolic compounds of extracts and fractions from *E. lindleyanum* DC was determined using Folin–Ciocalteu method and expressed as gallic acid equivalents. As shown in Table 1, BF had the highest phenolic content, which reached 78.62 mg of GAE/g.

**Table 1:** Total phenolics contents, IC_50_ values (DPPH radical and superoxide radical scavenging activities) of extracts and fractions of *Eupatorium lindleyanum* DC.

	Total phenolics (mg of GAE/g)	IC_50_ - DPPH radical (μg/mL)	IC_50_ - superoxide radical (μg/mL)
EtOH extract (EE)	25.35 ± 0.11 ^e^	n.a.	n.a.
PE fraction (PF)	6.70 ± 0.12 ^g^	n.a.	n.a.
EtOAc fraction (EF)	50.29 ± 0.51 ^d^	n.a.	177.9 ± 4.43 ^a^
BuOH fraction (BF)	78.62 ± 0.40 ^a^	74.99 ± 2.93 ^b^	102.61 ± 3.53 ^b^
Water fraction (WF)	17.28 ± 0.44 ^f^	n.a.	n.a.
Water extract (WE)	51.09 ± 0.68 ^c^	83.37 ± 0.44 ^a^	64.44 ± 0.72 ^c^
Water extract of residue (RWE)	56.60 ± 0.49 ^b^	70.56 ± 0.75 ^c^	54.09 ± 0.56 ^d^
BHA		3.03 ± 0.01 ^d^	26.77 ± 0.12 ^e^

Each value is presented as mean ± standard error (n = 3); Column wise values with same letter indicate no significant difference (*P* < 0.05); n.a.—values were not estimated.

Total phenolics were determined in greater amounts in BF than PF (6.7 mg of GAE/g), EF (50.29 mg of GAE/g) and WF (17.28 mg of GAE/g) prepared from the EE. Chua *et al.* [18] reported a similar result in that total phenolics could be concentrated by BuOH. The RWE (56.6 mg of GAE/g) contained a significantly higher amount of total phenolics than the WE (51.09 mg of GAE/g) and EE (25.35 mg of GAE/g) (P < 0.05). The results suggest that polar solvents were more suitable to extract the phenolic compounds from *E. lindleyanum* DC.

### 2.2. DPPH Radical Scavenging Activity

**Figure 1:**
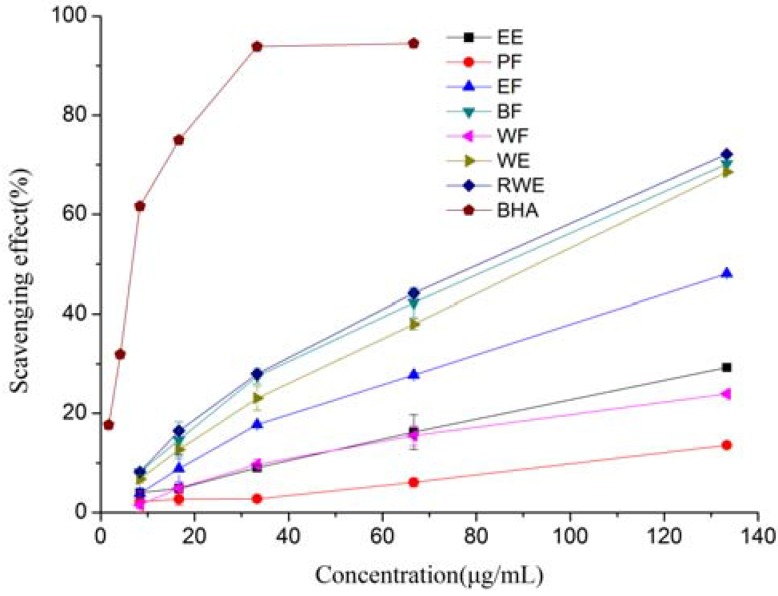
DPPH radical scavenging activity of extracts and fractions from *Eupatorium lindleyanum* DC. Results are mean ± SD (n = 3).

The DPPH radical is a stable nitrogen-centered free radical and has been extensively used to test the free radical scavenging ability of various samples. The DPPH scavenging activities of different extracts and fractions of *E. lindleyanum* DC were investigated. As shown in Figure 1 and Table 1, the radical scavenging activities of the test samples correlated well with increasing concentrations, and the highest activity was observed in the RWE, while BF and WE also showed good scavenging activities too. At the concentration of 133.33 μg/mL, the DPPH scavenging activity was found to be in the order of RWE (72.15%) > BF (70.17%) > WE (68.55%) > EF (48.05%) > EE (29.25%) > WF (23.94%) > PF (13.61%), respectively. It seemed that polar solvents were more suitable for extraction from *E. lindleyanum* DC of compounds with radical scavenging properties. The IC_50_ values of the BF, WE, RWE and BHA were 74.99, 83.37, 70.56 and 3.03 μg/mL, respectively. The IC_50 _values of EE, PF, EF and WF could not be calculated. When compared to BHA, all the tested samples showed significantly (P < 0.05) lower DPPH radical scavenging activity.

It could be observed that the content of total phenolics in the extracts and fractions showed good correlation with the DPPH scavenging activities (R^2^ = 0.9298), this result suggested that total phenolics were the major contributors to the DPPH scavenging activities of extracts and fractions of *E. lindleyanum* DC. Mariod *et al.* [19] also reported that the antioxidant activity of black cumin seedcake extracts and its fractions in DPPH assay was linearly correlated to its total phenolic compounds.

### 2.3. Superoxide Anion Radical Scavenging Activity

**Figure 2:**
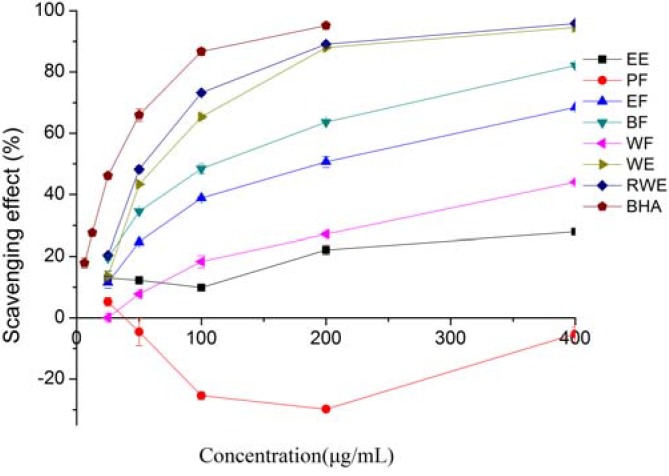
Superoxide radical scavenging activities of extracts and fractions from *Eupatorium lindleyanum* DC. Results are mean ± SD (n = 3).

Superoxide anion is a precursor to the formation in living systems of other ROS such as hydroxyl radical, hydrogen peroxide, or singlet oxygen. Therefore, superoxide anion scavenging is important for antioxidant activity. Figure 2 shows the superoxide anion radical scavenging activity of different extracts and fractions of *E. lindleyanum* DC. All the extracts and fractions except the PF one exhibited dose-dependent scavenging activity, suggesting that some of the extracts had a strong superoxide anion radical scavenging activity and could help prevent oxidative damage. The WE and RWE showed parallel scavenging effect, and had better inhibitory effects with 94.55% and 95.77% of inhibition at a concentration of 400 μg/mL, followed by BF (82.17%), EF (68.49%), WF (44.09), EE (27.92) and PE (−5.31%), respectively. The negative activity of PF suggested that some compounds in the PF could stimulate the superoxide anion radical, but more studies are needed to confirm this. The IC_50_ values, as shown in Table 1, were found to be 177.90, 102.61, 64.44, 54.09 and 26.77 μg/mL for EF, BF, WE, RWE and BHA, respectively. The IC_50_ values of EE, WF and PF could not be calculated. Although the scavenging activities of WE and RWE were slightly lower than that of BHA, they were much higher than those of EE, PF, EF, BF and WF. Several studies [20,21] have also reported that the water extract had a higher superoxide radical scavenging activity. The superoxide anion radical scavenging activity of different extracts and fractions of *E. lindleyanum* DC showed a moderate correlation with the total phenolics (R^2 ^=0.6739), this result suggested that most of the scavenging activities were due to the contribution of the total phenolics.

### 2.4. Total Reducing Power

**Figure 3:**
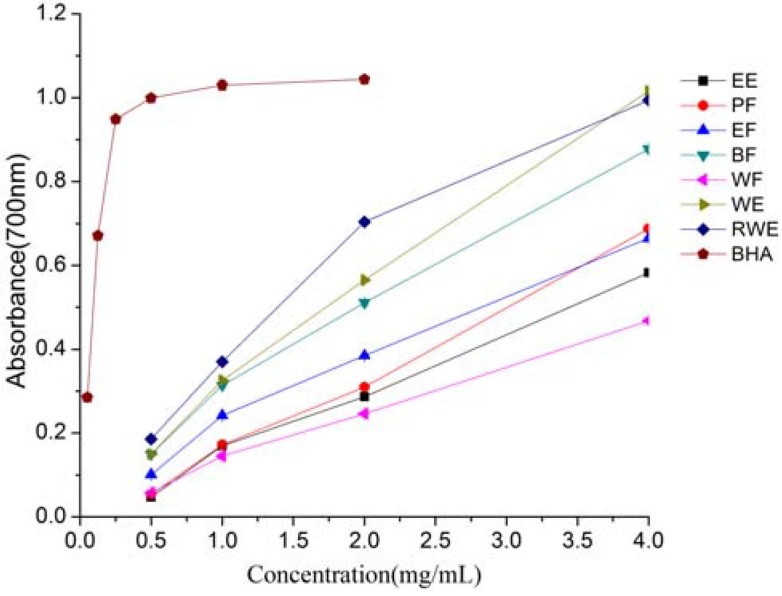
Total reducing power of extracts and fractions from *Eupatorium lindleyanum* DC. Results are mean ± SD (n = 3).

The reducing power of a compound may serve as a significant indicator of its potential antioxidant activity. The reductive activities were measured by the potassium ferricyanide reduction method, and quantified by absorbance measurement at 700 nm. Increased absorbance of the reaction mixture indicated increased reducing power. As shown in Figure 3, the reducing capacities of all the samples increased linearly with increasing concentration of the extracts. The same trend had also been reported by Chua *et al.* [18] for ethanolic extracts from the twigs of *Cinnamomum osmophloeum*. The reducing power of extracts and fractions at 4 mg/mL were as follows: WE (1.016) > RWE (0.994) > BF (0.878) > PF (0.688) > EF (0.665) > EE (0.583) > WF (0.468). However, when compared to BHA, all the samples exhibited weaker reducing power. This could be due to the presence of low relative concentrations of reducible substances in the extracts. A positive correlation was observed between the reducing power and the total phenolics (R^2^ = 0.7345). The results indicated that total phenolics were the major contributors to the total reducing power of extracts from *E. lindleyanum* DC.

### 2.5. Ferrous Ions Chelating Activity

**Figure 4:**
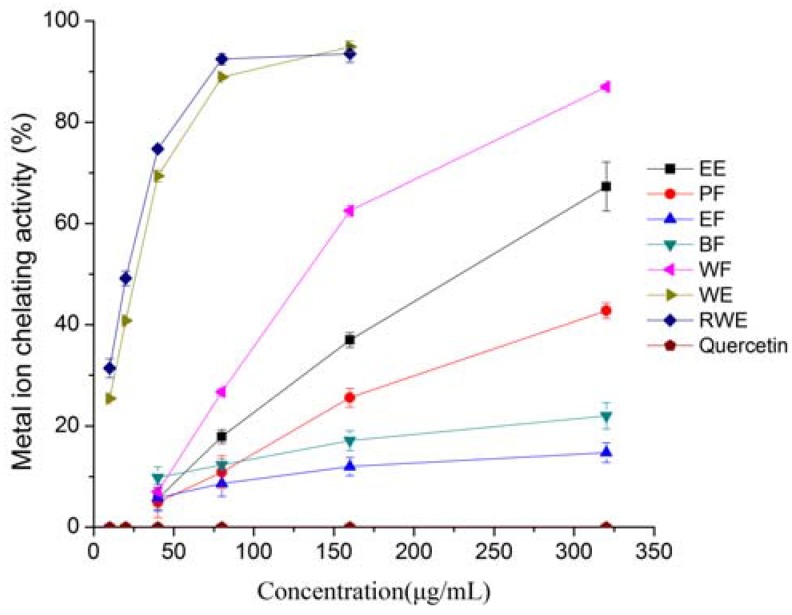
Metal chelating activities of extracts and fractions from *Eupatorium lindleyanum* DC. Results are mean ± SD (n = 3).

Bivalent transition metal ions play an important role as catalysts in oxidative processes, leading to the formation of hydroxyl radicals and hydroperoxide decomposition reactions via Fenton chemistry [22]. The chelating effects of the test samples on ferrous ions are shown in Figure 4. 

The WE and RWE showed significantly better iron chelation than other extracts or fractions, and exhibited a similar inhibitory effect. The chelating effects of the WE and RWE increased significantly with increasing concentration when the concentration below 160 μg/mL, after that, the chelating activity increased slowly as the sample concentration increased. On the other hand, the chelating effects of EE, PF, EF, BF and WF increased linearly with the increase in concentration, and at the concentration of 320 μg/mL, the chelating power was 67.32%, 42.78%, 14.73%, 22.02% and 87.00%, respectively, while for WE and RWE, the corresponding chelating effects were 94.93%, 93.54% at 160 μg/mL. Quercetin, however, did not show any chelating ability, which might be due to the presence of iron(II) chloride in the system that induces quercetin degradation; a similar result was reported previously by Tung *et al.* [23]. Evidently, the chelating ability decreased in the following order was WE, RWE > WF > EE > PF > BF > EF, this indicated that the compounds with strong chelating activity in this herb were polar, and it could be concluded that water was more suitable for extraction of such strong chelating ability substances from *E. lindleyanum* DC and that the *E. lindleyanum* DC extract had an effective capacity for iron binding if a proper extracting method is used. No significant correlation was found between the iron chelation ability of the extracts and total phenolics.

### 2.6. Analysis of Total Phenolics

Relatively few literature references describing the phenolic compounds of *E. lindleyanum* DC were available [10,24]. Tannins, chlorogenic acid, hyperoside, kaempferol and quercetin have been identified in *E. lindleyanum* DC extracts. Tannins are complex polyphenolic substances with great structural diversity and different water-solubilities. The content of tannins in WE, EE, RWE, BF and WF are shown in Table 2, the tannins levels in WE (26.47 ± 0.33 mg of GAE/g), RWE (27.51 ± 0.45 mg of GAE/g) were higher than that in EE (0.84 ± 0.16 mg of GAE/g), WF (2.81 ± 0.21 mg of GAE/g) and BF (0.73 ± 0.14 mg of GAE/g). In WE and RWE, most of the total phenolics are tannins (48.60% and 51.81%, respectively). In many studies [25,26], it has been stated that tannins are considerably more efficient antioxidants than lower molecular weight phenolic compounds. Tannins might thus be major contributors to the antioxidant activities of WE and RWE. Four phenolic compounds, namely chlorogenic acid, hyperoside, kaempferol and quercetin were identified and quantified by high performance liquid chromatography and the results presented in Table 2.

**Table 2:** Tannins and four phenolic compounds contents of extracts and fractions from *Eupatorium lindleyanum* DC.

	Tannins (mg of GAE/g)	chlorogenic acid (mg/g)	hyperoside (mg/g)	quercetin (mg/g)	kaempferol (mg/g)
EtOH extract (EE)	0.84 ± 0.16 ^d^	1.71 ± 0.05 ^c^	n.d.	0.50 ± 0.03 ^c^	0.31 ± 0.02 ^c^
PE fraction (PF)		n.d	n.d.	n.d.	0.39 ± 0.02 ^c^
EtOAc fraction (EF)		6.65 ± 0.04 ^a^	n.d.	1.62 ± 0.04 ^a^	0.98 ± 0.03 ^a^
BuOH fraction (BF)	0.73 ± 0.14 ^d^	3.53 ± 0.04 ^b^	n.d.	0.78 ± 0.03 ^b^	0.72 ± 0.04 ^b^
Water fraction (WF)	2.81 ± 0.21 ^c^	0.41 ± 0.02 ^f^	n.d.	n.d.	n.d.
Water extract (WE)	26.47 ± 0.33 ^b^	1.21 ± 0.03 ^d^	n.d.	n.d.	n.d.
Water extract of residue (RWE)	27.51 ± 0.45 ^a^	0.68 ± 0.02 ^e^	n.d.	n.d.	n.d.

Each value is presented as mean ± standard error (n = 3); Columnwise values with same letter indicate no significant difference (*P* < 0.05); n.d.—not detected.

**Figure 5:**
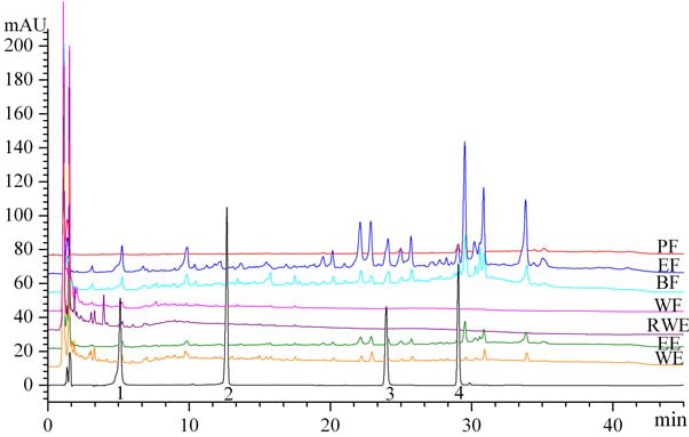
HPLC profile of extracts and fractions from *Eupatorium lindleyanum* DC. Peaks: 1, chlorogenic acid; 2, hyperoside; 3, quercetin;4, kaempferol.

The chromatograms with detector responses at 254 nm are shown in Figure 5. The EF (6.65 ± 0.04 mg/g) were found to have the highest chlorogenic acid content, followed by the BF (3.53 ± 0.04 mg/g), EE (1.71 ± 0.05 mg/g), WE (1.21 ± 0.03 mg/g), RWE (0.68 ± 0.02 mg/g) and WF (0.41 ± 0.02 mg/g). Hyperoside was not found in any of extracts or fractions, kaempferol was not found in RWE and WF, and quercetin was only found in EE (0.50 ± 0.03 mg/g), EF (1.62 ± 0.04 mg/g) and BF (0.78 ± 0.03 mg/g).

The total amounts of the four compounds obtained in EF and BF were 9.25 mg/g and 5.03 mg/g, respectively, significantly higher than that in the other extracts or fractions. The antioxidant activities of EF and BF are thus probably due to the higher contents of those phenolic compounds.

## 3. Experimental

### 3.1. Chemicals

1,1-Diphenyl-2-picrylhydrazyl (DPPH) was purchased from TCI Chemical Industry Co., Ltd (Tokyo, Japan) nitro blue tetrazolium (NBT), quercetin, gallic acid, and butylated hydroxyanisole (BHA) were purchased from Shanghai Jingchun Industry Co., Ltd (Shanghai, China). HPLC grade acetonitrile was purchased from the Jiangsu Hanbon Science & Technology Co., Ltd. (Huaian, China). Standards of chlorogenic acid, hyperoside, kaempferol and quercetin were purchased from Guizhou Di Da Technology Co., Ltd. (Guiyang, China). All the other chemicals employed were purchased from Sinopharm Chemical Reagent Co., Ltd (Shanghai, China). 

### 3.2. Plant Material

Plant material (*E. lindleyanum* DC) was purchased from Xuyi County of Jiangsu Province, China, and identified by one of the authors (Yuming Luo). The voucher specimens were deposited at the laboratory of the School of Life Sciences, Huaiyin Normal University.

### 3.3. Preparation of Samples

The air-dried powdered aerial parts of *E. lindleyanum* DC (10.0 kg) were soaked in 100 L of 95% EtOH for 3 days at ambient temperature. The extract was then filtered and collected. The same procedure was repeated four times. Then the collected ethanol extracts were combined, concentrated under reduced pressure on a rotary evaporator. The final yield of ethanol extract (EE) was 765.3 g. The last residue was extracted with 80 L of water at 100 °C for 30 min. The extract was filtered and collected. The extraction process was repeated three times, and the extracts were pooled together and filtered. The filtrates were concentrated under vacuum and the water extract of the residue (RWE) was thus obtained. The ethanol extract were extracted successively with petroleum ether, ethyl acetate, *n*-butanol, and water, then the solvent of every fraction was removed under reduced pressure. The final yields of petroleum ether fraction (PF), ethyl acetate fraction (EF), *n*-butanol fraction (BF), and water fraction (WF) were 214.65 g, 121.50 g, 68.21, and 275.93 g, respectively. Another 250 g dry powder of *E. lindleyanum* DC was placed in water (w/v, 1/8) and boiled at 100 °C for 30 min. After filtration, the plant materials were extracted twice in the same conditions. The water extract was collected, filtered, condensed under vacuum. The yield of water extract (WE) was 35.37 g. The ethanol extract (EE), petroleum ether fraction (PF), ethyl acetate fraction (EF), and *n*-butanol fraction (BF) were dissolved in DMSO prior to use, while the water extract of residue (WER) and water extract (WE) were dissolved in 60% DMSO.

### 3.4. Determination of Total Phenolics and Tannins

Total phenolic contents in the extracts and fractions were expressed as gallic acid equivalents (GAE) and determined according to the Folin-Ciocalteu method [27] with some modifications. Briefly, sample solution (0.1 mL, 4 mg/mL) and H_2_O (6 mL) were mixed with Folin-Ciocalteu reagent (0.5 mL). The contents were mixed well and kept for 5 min at room temperature followed by the addition of 20% aqueous sodium carbonate (1.5 mL). The solution was then immediately diluted to 10 mL with water and mixed thoroughly. After incubation at room temperature for 2 hours, the absorbance was measured at 760 nm against reagent blank. 

Tannin content in each sample was determined according to the method reported by Makkar *et al.* [28] with some modifications. Briefly, extract (1.0 mL, 4 mg/mL), was mixed with insoluble polyvinyl-polypirrolidone (PVPP, 100 mg) and water (1.0 mL), the contents were vortexed and kept at 4 °C for 15 min, and then the sample was centrifuged for 10 min at 3,000 rpm. The non-tannin phenolics content of the supernatant was determined as mentioned above. Tannin content was calculated as a difference between total phenolics and non-tannin phenolics content.

### 3.5. DPPH Radical Scavenging Activity

The free radical scavenging activities of the samples were determined using the DPPH method [29] with minor modifications. Briefly, sample solution (0.18 mL) of different concentrations (0.25–4 mg/mL) was mixed with 50 mM Tris-HCl buffer (1.62 mL, pH 7.4) and 0.1 mM DPPH in EtOH (3.6 mL). The well mixed solution was incubated for 30 min in darkness and at room temperature. The resultant absorbance was recorded at 517 nm. BHA was used as positive control. The percentage scavenging of the DPPH radical was calculated using the following equation:

DPPH scavenging effect (%) = (1 −As/Ac) ×100(1)

where Ac is the absorbance of the control reaction (containing all reagents except the test sample) and As is the absorbance of the test sample. The IC_50_ values were also determined as the concentrations of each sample required for 50% scavenging of DPPH radicals in the assay system.

### 3.6. Superoxide Anion Radical Scavenging Activity

The assay for superoxide anion radical scavenging activity was based on a riboflavin-light-NBT system [30,31] with some modifications. All solutions were prepared in 0.05M phosphate buffer (pH 7.8). The concentration of samples in the reaction mixture ranged between 25–400 μg/mL. The total volume of the reactant mixture was 5 mL and the concentrations of riboflavin, methionine and nitro blue tetrazolium (NBT) were 3 × 10^−3^, 10 and 0.1 mmol/L, respectively. The reaction solution was exposed to two 20W fluorescent lamps for 25 min and the absorbance was then measured at 560 nm. BHA was used as positive control. The reaction mixture without any sample was used as control. The superoxide anion radical scavenging activity was calculated as follows:

Scavenging effect (%) = (1−As/Ac) ×100(2)

where the As and Ac have the same meaning as in Equation (1).

### 3.7. Total Reducing Power

Total reducing power was estimated according to the method reported by Oyaizu [32] with some modifications. Various concentrations of extracts (0.5, 1.0, 2.0, 4.0 mg/mL, 1.0 mL) were mixed with phosphate buffer (2.5 mL, 0.2 mM, pH 6.6) and potassium ferricyanide (2.5 mL, 1%, w/v). After a 20 min incubation at 50 °C, the reaction was terminated by adding trichloroacetic acid (2.5 mL, 10%, w/v), and the mixture was centrifuged at 3,000 rpm for 10 min. An aliquot of the upper layer (2.5 mL) was mixed with water (2.5 mL) and FeCl_3_ (0.5 mL, 0.1%, w/v), and the absorbance was recorded at 700 nm against reagent blank. Increased absorbance of the reaction mixture indicated increased reducing power of the sample.

### 3.8. Ferrous Ions Chelating Activity

The chelation of ferrous ions by the extracts or fractions was estimated by the method of Dinis [33] with slight modificationw. Sample solutions (0.4 mL) with different concentrations (ranging between 10–320 μg/mL) and methanol (4.1 mL) were added to FeCl_2_ (0.1 mL, 2 mM). After 5 min incubation, the reaction was initiated by the addition of ferrozine (0.4 mL, 5 mM). The mixture was shaken vigorously and left standing at ambient temperature for 10 min. The absorbance of the reaction mixture was measured at 562 nm. Ferrous metal ions chelating activity was calculated as follows:

Metal ion chelating activity (%) = (1−As/Ac) × 100(3)

where the As and Ac have the same meaning as in Equation (1).

### 3.9. Analysis of Phenolic Compounds by High Performance Liquid Chromatography

The qualitative and quantitative analyses of the phenolic compounds in the extracts and fractions were performed at 254 nm using an Agilent HPLC (1260 series, Agilent Co., USA). Separations were performed on a ZORBAX Eclipse Plus C18 (4.6 mm × 100 mm, 3.5 µm) analytical column (Agilent Co., USA) operating at 30 °C. The solvent system was a mixture of 0.4% phosphoric acid(solvent A) and acetonitrile (solvent B), and the solvent gradient was as follows: 0–2 min isocratic 90% A, 2–5 min from 90% A to 85% A, 5–12 min from 85% A to 80% A, 12–20 min from 80% A to 75% A, 20–28 min from 75% A to 65% A, 28–38 min remained at 65% A, 38–44 min from 65% A to 90% A. A flow rate of 1 mL/min was used, and the injection volume was 25 µL. the WE and RWE were dissolved in 20% methanol, the other extracts and standards were dissolved in methanol. Identification of the phenolic compounds was carried out by comparing their retention times to those of standards. The phenolic compounds were quantified on the bases of their peak areas and the calibration curves of the corresponding standards.

### 3.10. Statistical Analysis

The data were reported as mean ± standard deviation (SD) (n = 3) and evaluated by one-way analysis of variance (ANOVA) followed by the Duncan’s multiple-range tests. Difference was considered to be statistically significant if P < 0.05.

## 4. Conclusions

To our knowledge, this is the first report concerning the antioxidant activities of extracts and fractions prepared from *E. lindleyanum* DC. Results from the present study showed that the WE and RWE had the strongest antioxidant captivities, and the BF also possessed high antioxidant abilities, except for the metal chelating activity, while the other extracts and fractions were relatively weak antioxidants. The content of total phenolics shows good correlation with DPPH radical scavenging activity, superoxide anion radical scavenging activity, and the reducing power. Although the tannins and four phenolic compounds were analyzed, the compounds responsible for the antioxidant activities of the extracts remain unclear, and further studies are urgently needed to better characterize, identify and isolate the compounds in the extracts, especially in the water extract and water extract of residue.
